# Does selection on horn length of males and females differ in protected and hunted populations of a weakly dimorphic ungulate?

**DOI:** 10.1002/ece3.2963

**Published:** 2017-04-17

**Authors:** Luca Corlatti, Ilse Storch, Flurin Filli, Pia Anderwald

**Affiliations:** ^1^ Freiburg Institute for Advanced StudiesUniversity of FreiburgFreiburgGermany; ^2^ Chair of Wildlife Ecology and ManagementUniversity of FreiburgFreiburgGermany; ^3^ Institute of Wildlife Biology and Game ManagementUniversity of Natural Resources and Life Sciences ViennaViennaAustria; ^4^ Swiss National ParkChastè Planta‐WildenbergZernezSwitzerland

**Keywords:** chamois, life history, longevity, *Rupicapra*, selective hunting, sexual selection

## Abstract

Weaponry in ungulates may be costly to grow and maintain, and different selective pressures in males and females may lead to sex‐biased natural survival. Sexual differences in the relationship between weapon growth and survival may increase under anthropogenic selection through culling, for example because of trophy hunting. Selection on weaponry growth under different scenarios has been largely investigated in males of highly dimorphic ungulates, for which survival costs (either natural or hunting related) are thought to be greatest. Little is known, however, about the survival costs of weaponry in males and females of weakly dimorphic species. We collected information on horn length and age at death/shooting of 407 chamois *Rupicapra rupicapra* in a protected population and in two hunted populations with different hunting regimes, to explore sexual differences in the selection on early horn growth under contrasting selective pressures. We also investigated the variation of horn growth and body mass in yearling males (*n *=* *688) and females (*n *=* *539) culled in one of the hunted populations over 14 years. The relationship between horn growth and survival showed remarkable sexual differences under different evolutionary scenarios. Within the protected population, under natural selection, we found no significant trade‐off in either males or females. Under anthropogenic pressure, selection on early horn growth of culled individuals showed diametrically opposed sex‐biased patterns, depending on the culling regime and hunters’ preferences. Despite the selective bias between males and females in one of the hunted populations, we did not detect significant sex‐specific differences in the long‐term pattern of early growth. The relationship between early horn growth and natural survival in either sex might suggest stabilizing selection on horn size in chamois. Selection through culling can be strongly sex‐biased also in weakly dimorphic species, depending on hunters’ preferences and hunting regulations, and long‐term data are needed to reveal potential undesirable evolutionary consequences.

## 1 Introduction

Darwin's (1871) theory of sexual selection provides an explanation for the evolution of extravagant differences between males and females in several phenotypical traits. Weaponry in male ungulates is one of the best‐studied secondary sexual characters (Emlen, 2008), whose shape and size are largely driven by selection operating through male–male competition over females (Andersson, 1994; Caro, Graham, Stoner, & Flores, 2003; Geist, 1966). Positive correlations between weapon size and male breeding success have been reported in several species such as red deer *Cervus elaphus* (Kruuk et al., 2002), bighorn sheep *Ovis canadensis* (Coltman, Festa‐Bianchet, Jorgenson, & Strobeck, 2002), Soay sheep *Ovis aries* (Robinson, Pilkington, Clutton‐Brock, Pemberton, & Kruuk, 2006), and Alpine ibex *Capra ibex* (Willisch, Biebach, Marreros, Ryser‐Degiorgis, & Neuhaus, 2015). In female ungulates, weapons—when present—are normally smaller than in males (Emlen, 2008) and they are thought to have primarily evolved through natural selection for defense against predators (Stankowich & Caro, 2009) rather than through sexual competition (Bro‐Jørgensen, 2007), although intrasexual selection can occasionally occur (Stankowich & Caro, 2009).

The development of secondary sexual characters may be energetically demanding (Solberg & Saether, 1993), and life‐history theory predicts that trade‐offs should constrain the simultaneous evolution of different—costly—traits (Stearns, 1992). Allocation of energy reserves and nutrients to weapon growth and maintenance is thus expected to impose fitness costs on individuals, for example, by reducing their survival (“trade‐off hypothesis”: Geist, 1971; Robinson et al., 2006). Costs should be greatest in males of ungulate species with high opportunity for sexual selection and marked sexual dimorphism in horn/antler size, because of the high energetic investment in weaponry. However, a negative correlation may occur only under food‐stress conditions (Stearns, 1992). Furthermore, individual heterogeneity may lead to a positive covariation between costly traits if individuals have different abilities to allocate their energy reserves (“individual quality hypothesis”), so that trade‐offs between fitness components may be difficult to observe (van Noordwijk & de Jong, 1986; Reznick, Nunney, & Tessier, 2000). In male ibex, for example, multilocus heterozygosity is positively related to body mass, which in turn positively affects horn growth (Brambilla, Biebach, Bassano, Bogliani, & von Hardenberg, 2015). Therefore, despite their high investment, ibex males do not show a trade‐off between horn growth and survival until old age (Bergeron, Festa‐Bianchet, von Hardenberg, & Bassano, 2008; Toïgo, Gaillard, & Loison, 2013). On the other hand, horns in female bovids are mainly shaped by natural selection (Stankowich & Caro, 2009), so that they are normally smaller than in males and the survival costs for weapon growth and maintenance are also expected to be lower.

Natural and sexual selection are not the only evolutionary pressures shaping weapon growth. Culling by humans, for example, is a major force that may impose constraints on the fitness trade‐offs of heritable secondary sexual characters (Allendorf & Hard, 2009; Festa‐Bianchet, 2003). The long‐term consequences of culling by humans on genetic and phenotypic characters depend on several cultural and bio‐ecological variables, such as hunters’ preferences, legal restrictions, and life‐history traits of the target species (Festa‐Bianchet, 2003, 2016). Given the opportunity, for example, hunters tend to selectively cull males with large horns or antlers (trophy hunting, Allendorf & Hard, 2009). If horn size is heritable and there is a strong relationship between mating success, age, and horn size, as in bighorn rams, fast‐growing individuals may be shot before they can reproduce, thus favoring males with smaller weapons (Coltman et al., 2003). Traill, Schindler, and Coulson (2014), using integral projection models, argued that the decline in traits such as body mass—which is strongly correlated to horn size—of the bighorn population is attributable mainly to demographic change and environmental factors, rather than to selective harvest. However, Chevin (2015) and Janeiro, Festa‐Bianchet, Pelletier, Coltman, and Morrissey (2017) recently showed that integral projection models, to date, are not appropriate to detect evolutionary changes. Hunting of females, on the other hand, is largely affected by the presence of offspring, either because hunters are reluctant to cull lactating females (Solberg, Loison, Saether, & Strand, 2000) or because they are penalized by hunting regulations (Rughetti & Festa‐Bianchet, 2014). The effects of culling females have mainly been explored from a demographic perspective (Jorgenson, Festa‐Bianchet, & Wishart, 1993; Rughetti & Festa‐Bianchet, 2014), while little is known about the effects of hunting on the sex‐specific natural patterns of weaponry growth.

Studies on the relationships between horn size and other fitness components under natural or anthropogenic selective pressures in ungulates have largely focused on males of highly dimorphic species (e.g., ibex, Toïgo et al., 2013; bighorn sheep, Coltman et al., 2003; Stone's sheep *Ovis dalli stonei*, Douhard, Festa‐Bianchet, Pelletier, Gaillard, & Bonenfant, 2016), for which survival costs (either natural or hunting related) are thought to be greatest. Similar relationships in both sexes in weakly dimorphic species have hardly been assessed, possibly because limited horn size is unlikely to impose major energetic costs, and because the opportunity for artificial selection may decrease with decreasing sexual size dimorphism (Mysterud, 2011). The northern chamois *Rupicapra rupicapra*, hereafter referred to as “chamois”, is a nearly monomorphic species widely distributed in the mountains of central Europe and the Near East (Corlatti, Lorenzini, & Lovari, 2011). Horns in chamois are unlikely to be under strong sexual selection, as they show limited sexual size dimorphism (Couturier, 1938) and do not seem to confer substantial advantages in male–male competition for mating (Corlatti, Caroli, Pietrocini, & Lovari, 2013; Corlatti et al., 2012). Furthermore, chamois horns are rather small, about 22 cm in males and 20 cm in females in the Alpine subspecies. Thus, their growth and maintenance are unlikely to represent major energetic costs for either sex. Surprisingly, however, Bleu, Loison, and Toïgo (2014) recently found a weak negative relationship between early horn growth and natural survival in female chamois, which may suggest covariation between early investment in horn size and other life‐history traits. Horn length in the first 2 years of life, for example, positively correlates with yearling body mass in both sexes (Rughetti & Festa‐Bianchet, 2010, 2011a) and with age of primiparity in females (Rughetti & Festa‐Bianchet, 2011a), thus providing a proxy of early energetic investment in male and female chamois. Furthermore, horns in both sexes show recovery growth (Corlatti, Gugiatti, & Imperio, 2015), a mechanism that likely limits the opportunity for artificial selection (Rughetti & Festa‐Bianchet, 2010) owing to the lacking development of conspicuous traits (Mysterud, 2011).

In this study, we investigate whether sexual differences in the relationship between early horn growth and longevity occur in the chamois under different selective scenarios. First we explore the trade‐offs between early horn growth and mortality in males and females within a protected population, under pressure of natural/sexual selection. We expect that trade‐offs between early horn growth and natural survival should be easier to detect in chamois than in highly dimorphic mountain ungulates. Individual heterogeneity, in fact, does not significantly affect the global pattern of horn growth in either sex in chamois: The absence of significant individual‐specific horn growth trajectories, in turn, may limit the occurrence of positive covariation between these fitness components (Corlatti, Gugiatti, et al., 2015). Specifically, we predict that the two sexes have similar trade‐offs: Females should show a weak negative relationship between early horn growth and survival (Bleu et al., 2014) because of the costs of early reproduction (Rughetti & Festa‐Bianchet, 2011a); males should show a weak negative relationship because horns are under weak pressure of sexual selection. We then investigate the relationship between early horn growth and survival under anthropogenic selection, in males and females culled within two hunted populations. We start from the null hypothesis that, given the weak sexual size dimorphism and the small horn size in chamois, the possibility for artificial selection should be low (Mysterud, 2011); hence, the relationship between early horn growth and age at shooting should be similar in either sex, and explore if this pattern holds true under different hunting regulations. Finally, we investigate the sex‐specific variation in early horn growth and the correlated yearling body mass over 14 years in one of the hunted populations. If anthropogenic selection is expressed through similar relationships between early horn growth and survival in the two sexes, we would expect similar temporal variation in phenotypic traits of males and females.

## 2 Materials and methods

### 2.1 Study areas and populations

**Figure 1:**
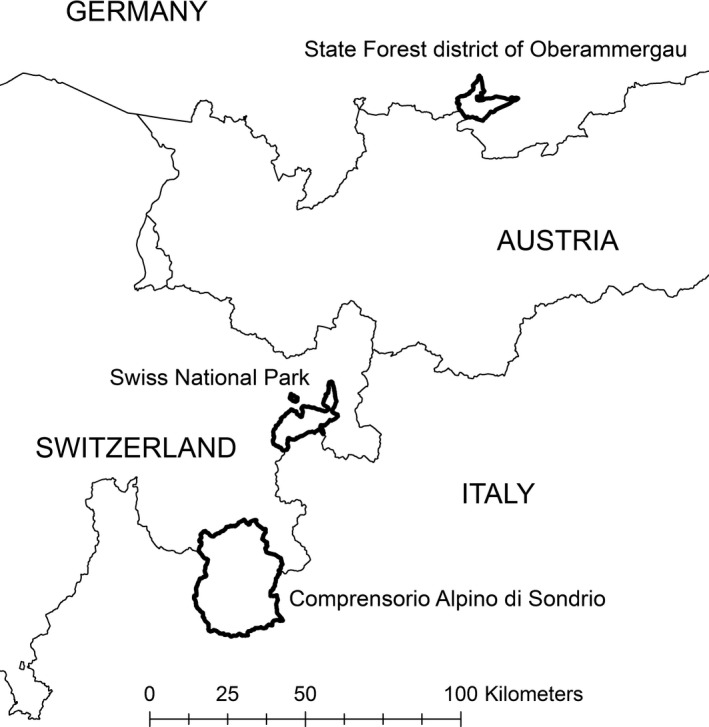
Map of the three study areas: Oberammergau (D), SNP (CH) and Sondrio (I)

Data from a non‐hunted chamois population were collected in the Swiss National Park (SNP) in southeast Switzerland in the central Alps (46°40′N, 10°12′E) (Figure 1). The SNP is a strictly protected nature reserve (IUCN category Ia) extending over 170 km^2^ between 1,400 and 3,200 m a.s.l. The area is characterized by an inner‐alpine continental climate with an average precipitation of 830 mm and annual mean temperature of 1°C (MeteoSwiss 2015). Overall chamois densities have varied between 6.1 and 9.9 individuals/km^2^ between 1990 and 2015.

Chamois culling data were collected in the hunting district of Sondrio (Comprensorio Alpino di Sondrio, hereafter Sondrio), and in the State Forest district of Oberammergau (hereafter Oberammergau). Sondrio extends over 780 km^2^ within the central Italian Alps (46°10′N, 09°52′E) (Figure 1), between 300 and 4,000 m a.s.l. and is about 50 km in a straight line from the SNP. Similarly to the protected area, it has an alpine‐continental climate, with a mean yearly rainfall of about 1,000 mm and mean yearly temperature of 1.5°C at 2,000 m a.s.l. (Pelfini & Belloni, 1990). The chamois population shows densities of about 6 individuals/km^2^, and the average annual culling plan is about 10% of the estimated population size. Since 1992, hunting quotas in Sondrio have been evenly distributed between males and females over three different age classes (1 year old, 2‐3 years old, and ≥4 years old). There are no specific restrictions for culling males within any age class, but hunters are penalized when culling lactating females up to 14 years of age. Oberammergau encompasses 100 km^2^ of the State Forest district of Oberammergau (47°33′N 11°06′E) (Figure 1) in its boundaries of 1985 in the Ammergauer Mountains in the Bavarian Alps, Germany (Storch, 1989). Elevations range from <900 m to >2,000 m a.s.l., and the climate is moist and temperate with a mean annual precipitation of >2,000 mm at 2,000 m a.s.l. (Storch, 1986). In Oberammergau, chamois hunting was conducted from 1 August to 15 December by State‐employed game wardens and guided guests; hunters highly valued the long horns of both male and female chamois as trophies, and there were no restrictions on lactating females (although hunters’ ethics would forbid shooting a lactating female before shooting her kid). In the year of data collection, 1985, game wardens conservatively estimated a summer population of at least 8.1 individuals/km^2^. Annual culls at that time amounted to around 100 individuals; the sex ratio in the yield was balanced, and age classes were evenly represented in females, but the hunting bag in males was skewed toward older ages (Storch, 1986, 1989).

### 2.2 Data collection

To analyze the sex‐specific relationship between survival and early horn growth, we collected information on the age at death and on the horn length in the first 2 years of life in chamois found dead within the SNP and culled in Sondrio and in Oberammergau. Within the protected area of the SNP, age at death reflects natural survival, while in Sondrio and in Oberammergau, age at shooting reflects artificial mortality. For all chamois, age at death was estimated by counting the number of horn rings (Schröder & von Elsner‐Schack, 1985). Sex identification for the SNP sample was based on horn morphology (Blagojević & Milošević‐Zlatanović, 2015; Couturier, 1938). Because in chamois it is difficult to distinguish horn growth in the first and second year of life, early horn growth was measured by means of a flexible ruler combining the first two segments (i.e., annulus L2, *cf*. Corlatti, Gugiatti, et al., 2015).

For the SNP sample, we used horn measurements of all animals found dead but in sufficiently good condition to enable determination of the year of death. Horn measurements of all individuals were catalogued with associated metadata (sex, age, location, and date the animal was found). Prior to analysis, we excluded all individuals that had not completed early horn growth (i.e., kids and yearlings). In the absence of knowledge about the causes of death, we also excluded from the analysis all chamois which showed adequate deposits of bone marrow fat (i.e., solid white or yellow fat) as they were unlikely to have died of starvation; their removal should reduce analytical issues that may occur when including animals that died because of casualties (e.g., avalanches, trauma). The SNP dataset consisted of *n *=* *125 individuals ≥2 years of age: 61 males and 64 females. The mean age at death in the SNP was 8.6 years in males and 9.4 years in females.

For Sondrio, we used the same dataset as Corlatti, Gugiatti, et al. (2015), which consisted of 194 individuals ≥3 years of age (101 males and 93 females) legally shot during the hunting seasons of 2009 and 2010. The age at death was set at x.5 years, because all individuals were shot between September and November, during about the sixth month of their current year of life. Mean age at harvest in the Sondrio sample was 5.7 years in males and 9.7 years in females. The L2 segment of the longest horn for each animal was measured during the annual trophy hunting exposition. From Oberammergau, we used a dataset consisting of 88 individuals ≥2 years of age (43 males and 45 females) legally shot during the hunting season of 1985, between August and December. The age at death and the L2 segment of the left horn were measured, as described above for the SNP and Sondrio, from skulls presented in preparation of the annual hunting trophy exposition at the State Forest Office at Oberammergau (Storch, 1986). The mean age at harvest in the Oberammergau sample was 7.8 years in males and 8.3 years in females. To avoid bias, we did not include horns with broken or worn‐out tips for any study area (SNP, Sondrio and Oberammergau); in Sondrio and in Oberammergau, during the annual trophy exposition, hunters were obliged by law to provide the clean skulls of all individuals culled over the hunting season.

Finally, we used data on horn length of yearling males (*n *=* *688) and yearling females (*n *=* *539) culled in Sondrio during 13 hunting seasons (September–November) between 1999 and 2013 (data were missing for the year 2007) to investigate sex‐specific variations in early horn growth (L2) over time. Because early horn growth may reflect variations in other life‐history traits (Rughetti & Festa‐Bianchet, 2010, 2011a), we also investigated sex‐specific variations in body mass for the same individuals.

### 2.3 Statistical analysis

To test for sexual differences in the relationship between longevity and early horn growth under different selective pressures, for each study site (SNP, Sondrio and Oberammergau), we fitted a multiple regression model with age at death as the response variable, while L2, sex, and the interaction between L2 and sex were fitted as predictors. In the SNP model, we also included year of birth as a random factor to control for possible cohort effects, while year of birth could not be included in the Sondrio and in the Oberammergau models because data were collected only in one or two hunting seasons. For each model, we centered the values of L2 to the mean because the intercept is interpreted as the expected value of the response variable when the predictors are set to zero, and this would not be realistic as L2 is always >0. For all three study sites, we first fitted linear models with normally distributed error terms and checked their goodness of fit through visual inspection of residuals. For the SNP linear mixed model and the Sondrio linear model, the residuals were normally distributed, but their variance increased with increasing predicted values of age at death. To improve linearity while accounting for variance heterogeneity, we thus fitted a generalized linear mixed model (GLMM) for the SNP, and a generalized linear model (GLM) for Sondrio, assuming a Gamma distribution function, in which the variance increases as the square of the mean. For the SNP model, we used a square root link, while for the Sondrio model, we used a log link, as the boxcox function suggested that λ = 0.5 and λ = 0 maximized their respective log likelihoods. For the Oberammergau hunting district, the goodness of fit of the linear model was satisfactory; thus, there was no need to fit GLMs. For all models, we calculated the percentile confidence intervals of regression estimates using a bootstrap procedure. We assessed the explanatory power of the linear model by *R* ^2^, of the GLM by calculating the pseudo‐*R* ^2^ values following Nagelkerke (1991), and of the GLMM by calculating the Ω_0_^2^ value following Xu (2003).

To test for sexual differences in the variation of yearling horn length and body mass in Sondrio between 1999 and 2013, we fitted generalized least square models (GLS) using a first‐order autoregressive structure to account for temporal autocorrelation in horn/mass metrics (*cf*. Douhard et al., 2016), setting horn length or body mass as response variables, and sex, year, and the interaction between sex and year as predictors. For both models, we included the Julian date of hunting as a covariate. When examining temporal trends in biological traits, however, results are strongly dependent on the level of analysis. To avoid reporting spurious results, we conducted a further analysis of temporal trends in male and female yearling horn length and body mass using ANODEV (Skalski, Hoffmann, & Smith, 1993). This procedure assesses the fit of the covariate model (i.e., year as a continuous variable, *M*_cov_) relative to that of both the baseline (i.e., constant, *M*_cst_) and the time‐dependent (i.e., year as a discrete factor, *M*_*t*_) models (*cf*. Grosbois et al., 2008 and Tafani, Cohas, Bonenfant, Gaillard, & Allainé, 2013). The *F* statistic obtained with ANODEV thus tests the null hypothesis that the covariate “linear year” has no impact on the variation of horn length and body mass in male and female yearlings, and it was defined as:

$$F_{\mathrm{cst}/\mathrm{cov}/t}=\frac{\frac{\text{Dev}\left(M_{\mathrm{cst}}\right)-\text{Dev}\left(M_{\mathrm{cov}}\right)}{J-1}}{\frac{\text{Dev}\left(M_{\mathrm{cov}}\right)-\text{Dev}\left(M_{t}\right)}{n-J}}$$

where Dev(*M*_cst_) is the deviance of the baseline (constant) model, Dev(*M*_cov_) is the deviance of the covariate model, and Dev(*M*_*t*_) is the deviance of the time‐dependent model. *J* is the number of parameters required to describe the relationship between yearling horn length/body mass and the covariate “linear year” for model *M*_cov_, while *n* is the number of parameters for model *M*_*t*_. We used linear models with normally distributed error terms to estimate the deviances. The *p*‐value for each *F*‐test was derived using *J − *1 and *n − J* as degrees of freedom. For each covariate model, we also calculated the *R* ^2^ following Skalski (1996):

$$R^{2}=\frac{\text{Dev}\left(M_{\mathrm{cst}}\right)-\text{Dev}\left(M_{\mathrm{cov}}\right)}{\text{Dev}\left(M_{\mathrm{cst}}\right)-\text{Dev}\left(M_{t}\right)}$$

*R* ^2^ quantifies the variation in average horn length and body mass in male and female yearlings accounted for by the covariate “linear year.”

All analyses were conducted using RStudio 1.0.136 (RStudio Team 2017) in R 3.3.2 (R Core Team 2016).

## 3 Results

**Figure 2:**
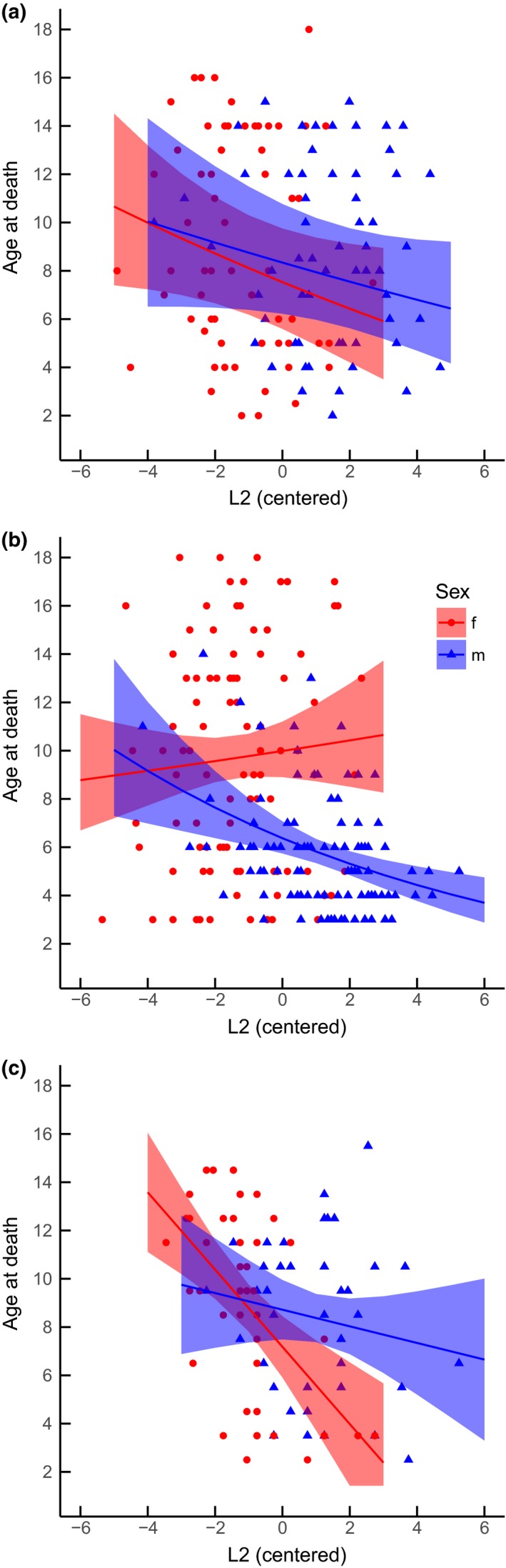
Models fitted to explain the sex‐specific relationships between early horn growth (L2) and age at death of chamois within (a) the protected population in the Swiss National Park and in two populations under different hunting regimes: (b) Sondrio, where hunters shot males of all ages, but had restrictions on lactating females; (c) Oberammergau, where hunters shot females of all ages, but primarily shot older males. Regression lines are reported with 95% confidence interval

In the protected area (SNP), age at death did not significantly decrease with increasing early horn growth in either males and females (Table 1, Figure 2a). The Ω_0_^2^ of the GLMM was 0.43. In the hunting area with restrictions on lactating females (Sondrio), the interaction between early horn growth and sex revealed a highly significant difference between the regression slopes for males and females, and only males showed a significant negative relationship between horn growth in the first 2 years of life and age at death (Table 1, Figure 2b). The pseudo‐*R* ^2^ value of the GLM was 0.32. The interaction between early growth and sex was also highly significant in the hunting area with no restrictions on lactating females (Oberammergau): Contrary to Sondrio, however, only females showed a significant negative relationship between early horn growth and survival (Table 1, Figure 2c). The *R* ^2^ value for the linear model was 0.15.

**Table 1:** Models explaining the relationships between age at death and early horn growth (L2), sex, and the interaction between L2 and sex in northern chamois within a protected population (SNP) and two hunted populations with different harvesting regimes: one with harvest restrictions on lactating females (Sondrio) and one without (Oberammergau). The table reports values of partial regression slopes (Estimate), standard errors (Std. Error), and percentile confidence intervals (95% CI) calculated using a bootstrap procedure. Regression slopes for L2 in males were calculated by refitting the models and setting males as the baseline sex using the “relevel” function in R. Significant predictors are shown in bold

	Estimate	Std. error	95% CI bounds	
			Lower	Upper
*SNP generalized linear mixed model*				
Intercept (females)	2.744	0.397	1.640	3.269
L2 (females)	−0.104	0.169	−0.438	0.288
L2 (males)	−0.070	0.136	−0.363	0.231
Sex (males vs. females)	0.144	0.437	−0.791	1.053
L2: Sex (males vs. females)	0.034	0.217	−0.467	0.475
*Sondrio generalized linear model*				
Intercept (females)	2.301	0.067	2.147	2.426
L2 (females)	0.021	0.034	−0.053	0.084
**L2 (males)**	−**0.091**	**0.019**	−**0.128**	−**0.055**
**Sex (males vs. females)**	−**0.449**	**0.082**	−**0.596**	−**0.268**
**L2: Sex (males vs. females)**	−**0.112**	**0.039**	−**0.192**	−**0.033**
*Oberammergau linear model*				
Intercept (females)	7.182	0.616	5.938	8.283
**L2 (females)**	−**1.600**	**0.315**	−**2.213**	−**1.028**
L2 (males)	−0.345	0.299	−0.921	0.261
Sex (males vs. females)	1.537	0.808	−0.032	3.082
**L2: Sex (males vs. females)**	**1.255**	**0.431**	**0.532**	**2.118**

**Table 2:** Generalized least square models fitted to explain sex‐specific variations in horn length (L2) and body mass of yearling chamois (*n *=* *688 males and *n *=* *539 females) harvested in Sondrio between 1999 and 2013. The table reports values of partial regression slopes (Estimate), standard errors (Std. Error), and 95% confidence intervals (CI). Regression slopes for Year in males were calculated by refitting the models and setting males as the baseline sex using the “relevel” function in R. Significant predictors are shown in bold

	Estimate	Std. error	95% CI bounds	
			Lower	Upper
Horn length (L2)				
Intercept (females)	115.110	35.078	46.357	183.863
**Year (females)**	−**0.052**	**0.018**	−**0.087**	−**0.017**
**Year (males)**	−**0.058**	**0.016**	−**0.089**	−**0.027**
Sex (males vs. females)	14.210	46.843	−77.602	106.022
Year: Sex (males vs. females)	−0.006	0.023	−0.051	0.039
Body mass				
Intercept (females)	165.487	53.709	60.217	270.757
**Year (females)**	−**0.076**	**0.027**	−**0.129**	−**0.023**
**Year (males)**	−**0.101**	**0.024**	−**0.148**	−**0.054**
Sex (males vs. females)	50.713	71.795	−90.005	191.431
Year: Sex (males vs. females)	−0.025	0.036	−0.096	0.046

**Figure 3:**
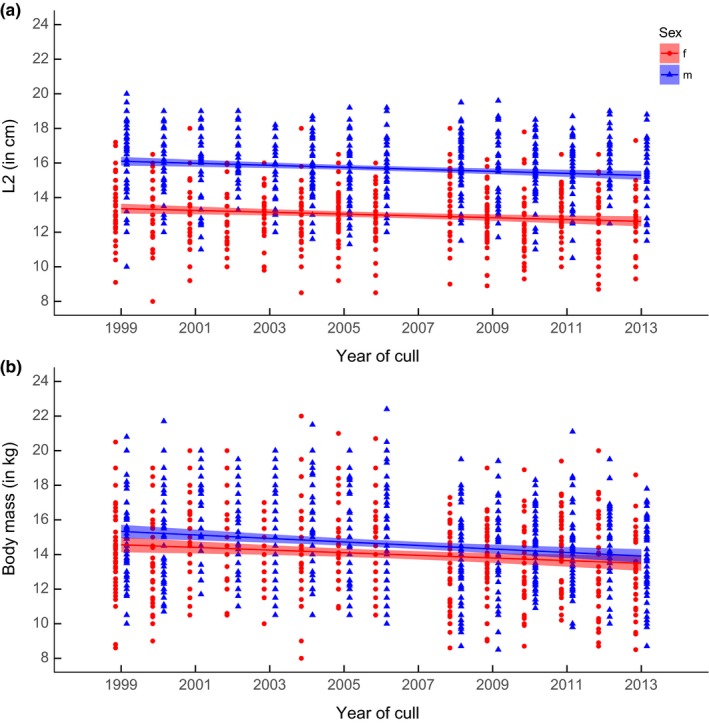
Generalized least square models of the sex‐specific variations in (a) horn length L2 and (b) body mass of yearling chamois (*n *=* *688 males and *n *=* *539 females) culled in Sondrio between 1999 and 2013. Regression lines are reported with 95% confidence intervals. Data are missing for the year 2007

The sexual variations in yearling horn length and body mass in Sondrio between 1999 and 2013 showed similar patterns: The interaction between year and sex was not significant in either GLS model (Table 1, Figure 3), while male and female yearlings showed a significant reduction in horn length and body mass over time (Table 1, Figure 3). The ANODEV procedure largely confirmed these last results: The covariate “linear year” had a significant negative impact on the temporal variation of yearling horn length in males (Est. = −0.051; *SE* = 0.015; *F*_cst/cov/t_ = 5.939; *p *=* *0.031; *R* ^2^ = 0.33) but not in females, although the *p*‐value was close to the significance level (Est. = −0.044; *SE* = 0.017; *F*_cst/cov/t_ = 4.689; *p *=* *0.051; *R* ^2^ = 0.28). The covariate “linear year” had a significant negative impact on the temporal variation of yearling body mass in both males (Est. = −0.099; *SE* = 0.021; *F*_cst/cov/t_ = 10.960; *p *=* *0.006; *R* ^2^ = 0.48) and females (Est. = −0.078; *SE* = 0.023; *F*_cst/cov/t_ = 6.037; *p *=* *0.030; *R* ^2^ = 0.33).

## 4 Discussion

Our results suggest the occurrence of remarkable variations in the relationships between early horn growth and survival in chamois under different selective scenarios. Within the protected population, under pressure of sexual/natural selection, we found statistically non‐significant negative trade‐offs between early horn growth and survival in both males and females. Conversely, we found major differences between the two sexes in the hunted populations, with early horn growth being under strong negative anthropogenic selection only in males where hunters were penalized for culling lactating females (Sondrio), and only in females, where hunters had no such legal restrictions, but spared males until old age (Oberammergau). Despite the strongly sex‐biased selection on early horn growth within the Sondrio population, yearlings did not show evidence for significantly different sex‐specific variation in horn length and body mass over time.

Within the protected population of the Swiss National Park males and females showed similar relationships between early horn growth and longevity, in line with the limited sexual difference in horn size (Couturier, 1938), skeletal size (Rughetti & Festa‐Bianchet, 2011b), and survival rates (Bocci, Canavese, & Lovari, 2010; Corlatti, Lebl, Filli, & Ruf, 2012; Gonzalez & Crampe, 2001), supporting the occurrence of similarly conservative life‐history strategies in the two sexes.

Our results are in slight contrast with the findings of Bleu et al. (2014) for a chamois population in the French Alps, where a marginal negative effect of early horn growth on female natural survival was detected. Bleu et al. (2014) discuss several hypotheses to explain this pattern, including energetic costs (Chirichella, Ciuti, Grignolio, Rocca, & Apollonio, 2013) and heat loss during winter (as observed in Barbary sheep *Ammotragus lervia*, Picard, Thomas, Festa‐Bianchet, & Lanthier, 1994). However, horns in female chamois are conspicuously shorter than in highly dimorphic bovids, and the—supposedly reduced—metabolic cost for their growth and maintenance seems unlikely to result in a detectable reduction in survival (*cf*. Poissant, Wilson, Festa‐Bianchet, Hogg, & Coltman, 2008). Different growth patterns may reflect variations in other life‐history traits. Horn length in the first 2 years of life, for example, positively correlates with yearling body mass and age at first reproduction in female chamois (Rughetti & Festa‐Bianchet, 2011a), so that a negative trade‐off between early horn growth and survival may indicate a reproductive cost (Bleu et al., 2014). The not statistically significant, albeit slightly negative, relationship between early horn growth and survival found in our study would thus suggest limited costs of early reproduction. Interestingly, recent studies did not find clear costs of reproduction at any age in female chamois, possibly owing to high individual heterogeneity in reproductive performance (Morin, Rughetti, Rioux‐Pasquette, & Festa‐Bianchet, 2016; Tettamanti, Grignolio, Filli, Apollonio, & Bize, 2015).

The trade‐off between early horn growth and survival in the SNP male sample was also statistically non‐significant. Although weapon size in male ungulates is mainly driven by sexual selection (Andersson, 1994), horn size in chamois does not seem to confer great advantages in male–male competition (although horn size may play a role in intrasexual competition in the Apennine subspecies *Rupicapra pyrenaica ornata*, Locati & Lovari, 1991); thus, they are unlikely to impose substantial energetic costs. Notwithstanding the lack of statistical significance, the effect size of the trade‐off may have important consequences on the life history traits of the chamois. Should the negative effect of early horn growth on male survival be confirmed in other protected populations of the northern species, it is possible that male chamois with relatively longer horns have some limited reproductive advantage over shorter‐horned individuals, and that the increased costs of reproduction may cause a slight reduction in their survival probabilities. The effect size of such a trade‐off, however, should be small; it also remains unclear to what extent potential survival costs are due to early growth in horn size or to related life history traits such as early growth in body mass. It should also be noted that, although we excluded from our analysis horns with clear signs of wear, the weak negative relationship between early horn growth and survival in either sex may be partly explained by tip deterioration occurring at old ages.

The patterns observed in the hunted individuals largely differ from the natural trade‐offs observed in the SNP. In the absence of data on individuals that died of natural causes within the hunted populations, it is difficult to determine to what extent these patterns are a direct consequence of hunting or the result of actual variations in natural mortality of individuals across the study populations. In fact, sex‐specific adult survival in ungulates can differ depending on environmental conditions. Typically, however, the costs of secondary sexual characters tend to increase with increasingly harsh environmental conditions: Mortality, for example, is greater in males than in females of dimorphic species when populations inhabit food‐limited environments (Toïgo & Gaillard, 2003). We might therefore expect that in harsh environments, as early horn size (thus early body mass) increases, males could suffer higher mortality than females, for example, because of relatively higher energy expenditure during the rutting season. Our study sites show similar values of chamois density, and two populations (SNP and Sondrio) live in similar habitats. Furthermore, food availability conditions in Oberammergau are likely to be more favorable than in the SNP and in Sondrio. Under pressure of natural selection, we would thus expect the patterns of selection on early horn growth to be similar between males and females also in the hunted populations. If so, we suggest that culling, rather than environmental variations, is likely to be a major driver of the observed sexual differences in the relationships between early horn growth and survival in our study populations.

Ungulate hunting is generally trophy oriented and hunters preferably cull older males with longer horns, but the occurrence of weak sexual size dimorphism and small horn size should limit the opportunity for artificial selection (Mysterud, 2011). Our data suggest that artificial selection on horn length can be noticeable even in a poorly dimorphic species and that this ability interacts with hunters’ preferences and hunting regulations to shape the sex‐specific patterns of the relationship between early horn growth and survival. When restrictions are imposed on lactating females, but not on males, as in Sondrio, males show a negative relationship between early horn growth and survival. In the absence of data on males that died of natural causes in this population, it is difficult to assess whether the negative value of the relationship is due to trophy hunting or if it reflects male availability due to natural mortality (although Figure 2 shows weak differences among populations, possibly suggesting limited opportunity for artificial selection on male horn size, *cf*. Rughetti & Festa‐Bianchet, 2010). Females, on the other hand, showed a significantly different pattern to males: Long‐horned females tend to give birth at an earlier age than short‐horned individuals (Rughetti & Festa‐Bianchet, 2011a), thus reducing the opportunity for trophy hunting on young individuals if lactating females are protected. This selective regime, if combined with the adoption of hunting quotas for different age classes—which favors a high percentage of old (11+ years) non‐lactating females in the hunting bag (about 30%–40% in Sondrio)—may relieve the culling pressure on young individuals with high reproductive value, possibly offering advantages over the random hunting regime proposed in the model by Rughetti and Festa‐Bianchet (2014). Given the possibility, however, hunters’ selectivity on females may also be oriented toward trophy hunting, as in Oberammergau, leading to a diametrically opposed sex‐biased relationship between early horn growth and survival. Although we do not have information on females that died of natural causes in Oberammergau, confidence intervals in Figure 2 suggest that the slope for females in this area is significantly steeper than in the other populations, supporting the opportunity for trophy hunting also in female chamois. This result supports the suggestion by Mysterud (2012) that females may become a target for trophy hunters when sexual dimorphism is small, as in oryx (*Oryx gazella*) and eland (*Taurotragus oryx*). In chamois, the fact that females tend to live in larger groups than males (Krämer, 1969) likely increases the possibility for hunters to select individuals with longer horns (Mysterud, 2011). Given the positive correlation between early horn growth and age of primiparity (Rughetti & Festa‐Bianchet, 2011a), and the occurrence of individual heterogeneity in reproductive performance (Morin et al., 2016), the possibility of trophy hunting on female chamois deserves to be taken into account when evaluating the demographic effects of different selective regimes, as it is likely to deeply affect population dynamics (Mysterud, 2012). The unexpected lack of a relationship between early horn growth and male age at harvest in Oberammergau may be partly explained by the small sample size. Nonetheless, with similar sample size, females showed a significant negative relationship, and it seems plausible that the lower effect size in males may be due to the skewed distribution toward older individuals in the hunting bag (mean age of males culled in Oberammergau: 7.8 years; in Sondrio: 5.7 years). In Oberammergau, in fact, game wardens spared males for shooting them at an older age (I. Storch, pers.com.).

In Sondrio, hunting restrictions on lactating females have been adopted since 1992. Despite the sex‐biased hunters’ selectivity, however, the GLS models offer no support for sex‐biased variation in yearlings’ horn length or body mass over time. The limited investment in horn length that chamois make in their first years of life is likely to reduce the overall variance in early growth compared to large‐horned species, and the length of our sampling period may be insufficient to detect potential consequences of selective culling. Furthermore, the mechanisms of harvest selectivity are complex (Mysterud, 2011) and there may be several reasons why the occurrence of negative selection on males, but not on females, with larger horn growth did not result in different sex‐specific patterns of variation in horn length and body mass over time. Assuming that horn size is a heritable trait in chamois, for example, it is possible that the lack of negative selection on females’ early horn growth in Sondrio may mitigate the possible effect of trophy hunting on males. Furthermore, the opportunity for artificial selection on chamois trophy size may be limited by the behavioral characteristics of the species. Selective culling should have greater evolutionary effects “if traits that determine trophy quality confer a fitness advantage at an advanced age” (Douhard et al., 2016): In chamois, however, horn size is under weak pressure of sexual selection and fitness advantages of growing larger weapons are probably limited. In addition, male chamois reach asymptotic horn size and body mass at about 5 years of age (Bassano, Perrone, & von Hardenberg, 2003) and they can successfully sire offspring at the age of 6 (Corlatti et al., 2015), but in hunted populations, the age at first reproduction may be lowered because of excessive culling of adults (if older males are shot, younger males will breed, Bon, Gonzalez, Bosch, & Cugnasse, 1992). Therefore, despite a possible negative selection by hunters, it is plausible that in Sondrio longer‐horned males can successfully reproduce before being shot, eventually reducing potential negative long‐term effects of trophy hunting. Evolutionary effects of trophy hunting can also be influenced by culling pressure (Douhard et al., 2016; Mysterud, 2011), and it would be interesting to investigate the long‐term variation of fitness‐related traits under different scenarios.

Finally, the GLS models suggest that yearling horn size and body mass showed a significant reduction over time: These results are largely confirmed by ANODEV, although horn length in yearling females returned a *p*‐value of 0.051. Although Coltman et al. (2003) found evidence for a decline in horn size in bighorn rams over 3 decades, and Douhard et al. (2016) found a similar decline in Stone's sheep over 4 decades, owing to evolutionary effects of trophy hunting, further analyses are needed to clarify the role of variables underlying this trend in chamois. The statistically non‐significant variation of horn length in females, as opposed to males, might indeed suggest the occurrence of diverse evolutionary pressures acting on the two sexes, although sex‐specific trends do not appear significantly different. It remains unclear, however, to what extent these trends depend on contrasting hunting regimes: In fact, similar patterns have recently been observed in other chamois populations on the Alps, possibly owing to reduced food availability or increasing competition due to climatic changes (Mason, Apollonio, Chirichella, Willis, & Stephens, 2014; Rughetti & Festa‐Bianchet, 2012).

## 5 Conclusion

The lack of data on natural mortality in the hunted populations, and the lack of replication in our study impose some caution on any true inference regarding the factors that drive the observed differences, due to variations in space, time, and hunting regime. It should be noted, however, that in hunted populations, it would be difficult to collect data on individuals that died of natural causes, as hunting normally accounts for a large part of the mortality events; furthermore, true replicates are difficult to obtain, as chamois hunting management is extremely variable both across and within countries (Damm & Franco, 2014), and it often varies over time. Notwithstanding these caveats, we argued that environmental differences are unlikely to fully account for the observed differences among sites, and that human hunting may play a major role in shaping the different patterns of relationship between early horn growth and survival. The unbiased sex‐specific patterns observed within the protected population suggest that, under natural selection, the “trade‐off hypothesis” rather than the “individual quality” hypothesis might be supported in male and female chamois. Further data, however, are needed to support this suggestion. If confirmed, larger horn size may be naturally counter‐selected in both sexes, as already suggested by the occurrence of a mechanism of compensatory growth (Corlatti, Gugiatti, et al., 2015; Rughetti & Festa‐Bianchet, 2010). Under pressure of anthropogenic selection, although the weak sexual size dimorphism should limit the hunters’ ability to select individuals, chamois may show diametrically opposed sex‐biased patterns of relationship between early horn growth and survival, depending on hunters’ preferences and on hunting regulations. Interestingly, although trophy hunting has been largely investigated in male ungulates, our data suggest that it can also affect life‐history traits of females.

Further studies are needed to clarify the mechanisms underlying the sex‐specific relationship between early horn growth and survival under different selective pressures, especially in terms of covariation between early horn growth and other life‐history traits. Our results support Rughetti and Festa‐Bianchet's (2010) suggestion that the behavioral, morphological, and ecological features of the chamois likely limit the opportunity for artificial selection on the patterns of horn growth in this species (see also Festa‐Bianchet, 2016). However, we suggest that hunting deserves to be taken into account as an evolutionary pressure that may shape the pattern of weapon growth also in weakly dimorphic species, and long‐term data collection on early horn growth and age at death are needed to reveal potential undesirable evolutionary/demographic effects of different culling regimes not only on males, but also on females.

## Authors’ contributions

LC conceived the idea, collected the Sondrio data, did the statistical analyses, and wrote the first draft of the manuscript. IS collected the Oberammergau data and participated in writing up and revising the manuscript. FF provided the data for the SNP and participated in revising the manuscript. PA took part in the statistical analysis and participated in writing up and revising the manuscript. All authors contributed critically to the drafts and gave final approval for publication.

## Conflict of interest

None declared.
